# Medical students’ attitudes and perceived competence regarding medical cannabis and its suggestibility

**DOI:** 10.1186/s12909-024-05089-8

**Published:** 2024-02-15

**Authors:** Tatjana Denneler, Moritz Mahling, Sabine Hermann, Andreas Stengel, Stephan Zipfel, Anne Herrmann-Werner, Teresa Festl-Wietek

**Affiliations:** 1 Deanery of Students’ Affairs, Faculty of Medicine, University of Tuebingen, Tuebingen, Germany; 2 TIME– Tuebingen Institute for Medical Education, University of Tuebingen, Tuebingen, Germany; 3 Department of Diabetology, Endocrinology, Nephrology, Section of Nephrology and Hypertension, University Hospital Tuebingen, Tuebingen, Germany; 4 University Department of Anaesthesiology and Intensive Care Medicine, University Hospital of Tuebingen, Tuebingen, Germany; 5 Department of Psychosomatic Medicine and Psychotherapie, University Hospital of Tuebingen, Tuebingen, Germany; 6 German Center for Mental Health (DZPG), site Tuebingen, Tuebingen, Germany; 7 Center for Internal Medicine and Dermatology, Deparment of Psychosomatic Medicine, Charité-Universitätsmedizin Berlin, Corporate Member of Freie Universität Berlin and Humboldt-Universität zu Berlin, Berlin, Germany

**Keywords:** Attitudes, Cannabis, Chronic pain, Competence, Influence, Medical cannabis, Medical education, Medical students, Pain medicine, Students

## Abstract

**Introduction:**

The global trend of legalizing medical cannabis (MC) is on the rise. In Germany, physicians have prescribed MC at the expense of health insurers since 2017. However, the teaching on MC has been scant in medical training. This study investigates medical students’ attitudes and perceived competence regarding MC and evaluates how varying materials (videos/articles) impact their opinions.

**Methods:**

Fourth-year medical students were invited to participate in the cross-sectional study. During an online session, students viewed a video featuring a patient with somatoform pain discussing her medical history, plus one of four randomly assigned MC-related materials (each an article and a video depicting a positive or negative perspective on MC). Students’ opinions were measured at the beginning [T0] and the end of the course [T1] using a standardized questionnaire with a five-point Likert scale. We assessed the influence of the material on the students’ opinions using paired-sample t-tests. One-way analysis of variance and Tukey post-hoc tests were conducted to compare the four groups. Pearson correlations assessed correlations.

**Results:**

150 students participated in the course, the response rate being 75.3% [T0] and 72.7% [T1]. At T0, students felt a little competent regarding MC therapy (M = 1.80 ± 0.82). At T1, students in groups 1 (positive video) and 3 (positive article) rated themselves as more capable in managing MC therapy $(\text{t}\left(28\right)=-3.816,\text{p}<0.001; \text{t}\left(23\right)=-4.153,\text{p}<0.001)$, and students in groups 3 (positive article) and 4 (negative article) felt more skilled in treating patients with chronic pain $(\text{t}\left(23\right)=-2.251,\text{p}=0.034;\text{t}\left(30\right)=-2.034;\text{p}=0.051)$. Compared to the other groups, group 2 students (negative video) felt significantly less competent. They perceived cannabis as addictive, hazardous and unsuitable for medical prescription.

**Discussion:**

This study showed that medical students lack knowledge and perceived competence in MC therapy. Material influences their opinions in different ways, and they seek more training on MC. This underlines that integrating MC education into medical curricula is crucial to address this knowledge gap.

## Introduction

In 2020, it was estimated that 209 million people worldwide were actively consuming cannabis. These numbers have been increasing [1]. Besides “recreational” cannabis, the number of countries legalizing medical cannabis (MC) is rising [2]. Since early 2017, it has been legal for doctors in Germany to prescribe MC after a decision on a by-case basis at the expense of health insurers. This law gives the treating physicians a wide margin of discretion regarding which patients they recommend, offer and/or prescribe therapy with MC. (§ 31 sub-paragraph 6 phrase 1 SGB V (Germany)).

### Medical cannabis and chronic pain

MC comprises medicinal products that contain cannabinoids and are used to treat diseases or their accompanying symptoms [3]. In Germany, different dosage forms of MC are available, e.g., dried cannabis flowers or dronabinol drops or capsules. The (adverse) drug effects are manifold and not sufficiently predictable [4].

However, patients have high hopes regarding this new treatment option [5–8]. A study by Rochford et al. (2019) showed that four out of five patients with chronic pain felt that MC could have health benefits for them [9]. Chronic pain is an independent medical condition. It is referred to when a patient has been experiencing pain for at least three months [10]. The main characteristic of somatoform disorders is the occurrence of physical symptoms without a biological correlate or organically detectable cause [11]. The guideline for somatoform pain disorders states that MC is not indicated “for purely functional complaints” [12].

Since the legalization of MC in Germany, 191.148 prescriptions of MC were filled from January to June 2022 alone [13]. As of March 2022, the most common causes for prescribing MC were chronic pain (76.4%),malignomas (14.5%),spasticity (9.6%),multiple sclerosis (5.9%) nd cachexia/wasting (5.1%) [14] There is only one “best-practice” guideline for prescribing MC in pain medicine published by the German society for pain medicine and no evidence-based guideline [15].

### MC in medical studies

With the entry into force of the new law, it is the responsibility of the attending physician to decide on the treatment with MC. Only the first prescription requires a by-case approval from the health insurance company.

In contrast, teaching about MC has had little or no place in medical studies [16, 17]. Medical students do not feel adequately prepared concerning the topic of MC. They are insecure regarding counselling patients about MC [16, 17] and the legal framework of prescribing MC [18]. Furthermore, they demand more teaching about MC [16–18].

A study by Gardiner et al. (2019) found that medical students have a positive attitude towards the use of MC in general but raised concerns regarding the risks of MC and their own competence with MC [18]. These concerns about their competence confirm a study which explored how patients with epilepsy gain information about MC. The study showed that only 10.9% o patients were informed about MC by their treating doctor. The majority gained their information by consulting non-medical sources like the Internet (36.7%) or family/friends (24.7%) [8] When asked how practicing physicians gain information about MC, 57% name the media/news, whilst only 23.6% statd lectures as a source of information [19]. Literature on teaching MC is still lacking in Germany.

### Aim of the study

Due to the novelty of the law and the previously unassigned medical responsibility for deciding on an indication for treatment with MC, the use of MC is not adequately covered in the current regular curriculum [20]. Previous studies showed that medical students feel insecure regarding MC and demand more training on it [16–18]. Therefore, this study aimed to investigate medical students’ attitudes and perceived competence regarding the use of MC.

## Materials and methods

### Study design

This cross-sectional study– focusing on the attitudes of medical students towards the use of cannabis in pain medicine– was conducted at the Medical Faculty of the Eberhard-Karls University of Tübingen, Germany. Fourth-year medical students were invited to participate in the study during their regular mandatory course in pain medicine (“QB 14”: cross-sectional course No. 14, see 2.4). The study took place in a seminar of the Q14 course as an online meeting due to the pandemic situation using Zoom© in summer term 2021 and winter term 2021/2022 (i.e. from April 2021 to February 2022).

### Ethics

The study received ethics approval from the Ethics Committee of Tübingen Medical Faculty (no. 578/2021B02). Alignment with the rules of the Helsinki declaration was ensured by obtaining informed consent from all participants and by guaranteeing voluntary participation, confidentiality and freedom to withdraw from the study at any point in time without any explanation. Students did not receive any reimbursement for participating. All responses and data were kept anonymous.

### Measurements

A quantitative questionnaire consisting of 27 items was used. Twenty items were rated on a five-point Likert scale ranging from 1 (“I strongly disagree”) to 5 (“I strongly agree”) to assess the student’s knowledge about and judgement of therapy with cannabinoids or opioids. Of these 20 items, 12 items are part of the Drug Attitude Scale (DAS). More precisely, two subscales– the opioid scale and the cannabis scale of the DAS– were used. The DAS measures personal attitudes and behaviour regarding substance use and abuse. It consists of 60 items that evaluate attitudes towards ten substances/drugs [21]. There was a transcription error in our translation of the original statement in item 16. Therefore, the opioid scale used was reduced by one item.

The perception of the student’s own competence was assessed using four items rated on a five-point Likert scale ranging from 1 (“not at all”) to 5 (“very competent”). To measure the student’s perception, questions such as “How competent do you feel in dealing with patients with chronic pain disorders (CPD)?” or “How competent do you feel in managing medical cannabis therapy?” were used.

Two more items assessed the need for training on MC in medical school and in continuing medical education for doctors. The last item was only used at T1 to assess the perceived influence of the seminar on the student’s opinion on MC.

### Procedure

**Fig. 1:**
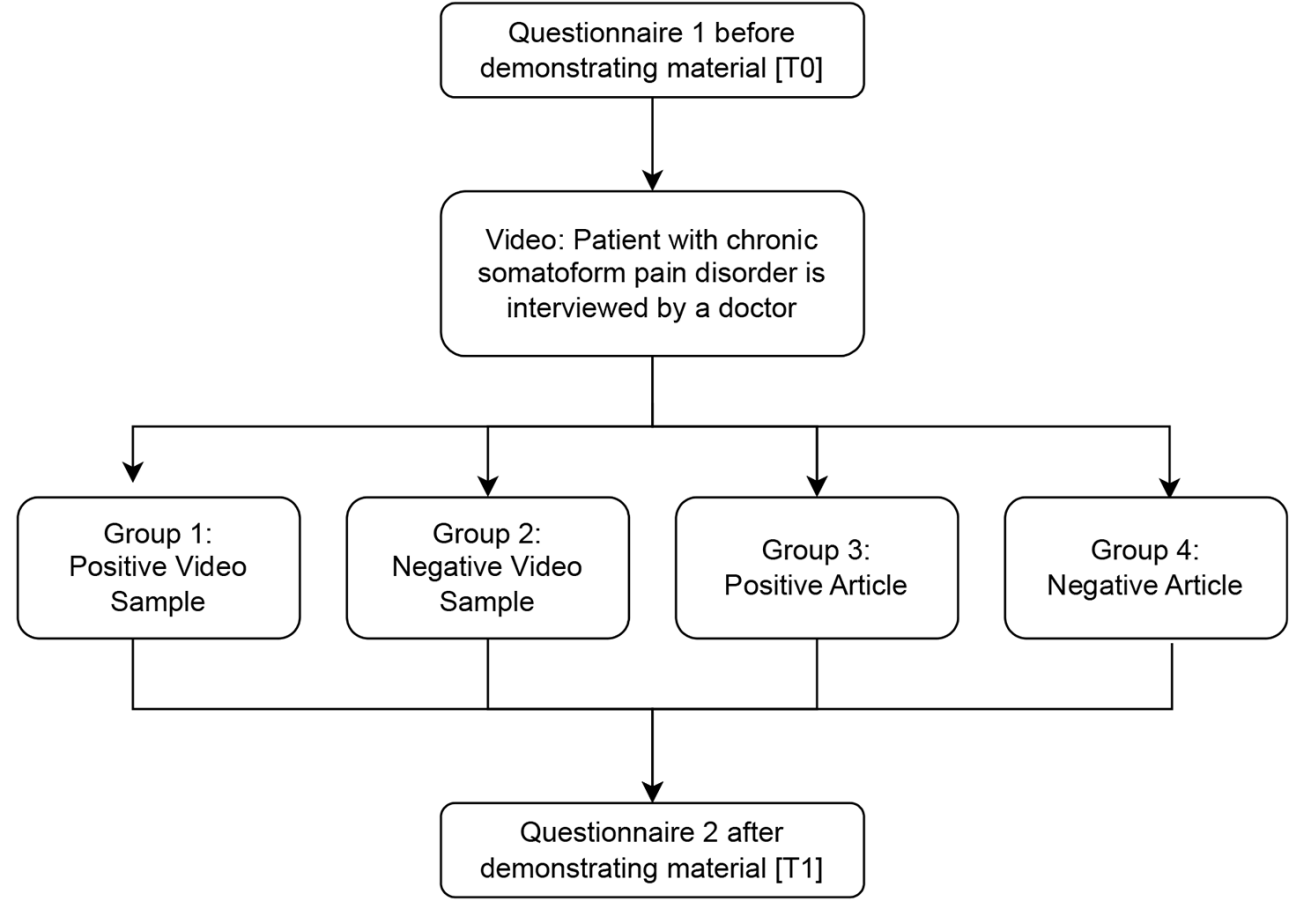
Procedure of the study

In the QB 14 course, fourth-year medical students learn about pain medicine including how to diagnose and treat patients with chronic pain, such as migraines. It’s an interdisciplinary course in which anaesthesiologists, neurosurgeons and psychosomatic specialists contribute. It consists of seven lectures taught by the different disciplines each lasting 90 min and two seminars each lasting 60 min. The study took place in one of the seminars which was always conducted by the same lecturer (an expert in psychosomatic medicine). In total the study was conducted during ten seminars where 9–13 students participated. To measure the impact of the session on the students’ attitudes, they were asked to fill out the survey at the start of the session [T0] and after the session [T1], as illustrated in Fig. 1. Discussions on the topic were only allowed after completing the second survey at T1. After completing the questionnaire at T0, students, first, viewed a video in which a physician took the medical history of a patient with a chronic pain disorder with psychological and somatic factors in a psychosomatic day-care clinic.

Afterwards, each student was simply randomized by using tables with random numbers to one of the following groups with the corresponding material and shown one of the following materials:

- Group 1 and 2 saw a video of a simulated patient-physician-encounter. The simulated patient came in for a follow-up regarding his experience with the newly prescribed MC for the chronic pain he suffers from. In this video, the main focus is not on the indication for MC but on how the patient was dealing with the MC therapy and what experiences he made. In group 1, the patient stated that his pain level has been reduced because of the MC therapy to such an extent that his previous opiate intake could be reduced. The video lasted 1 min 20 s and was constructed and recorded for the purpose of this study. In group 2, the patient stated that he didn’t experience any change in his pain levels and couldn’t reduce his previous opiate intake. On the contrary, he felt less like himself and stopped taking the prescribed CAM. The video lasted 1 min 20 s and was constructed and recorded for the purpose of this study.
- Group 3: The students were given an article to read in which the use of medical cannabis was predominantly reported positively [22].
- Group 4: The students were presented with a newspaper article in which the use of medical cannabis was reported in a predominantly negative way [23].

### Data analysis

The Kolmogorov-Smirnov test showed no normal distribution of the data. However, recent simulation studies prove that the normal distribution of the data is not an important requirement for evaluation with parametric tests [24]. Moreover, it has been shown that from a sample size > 30, the test of normal distribution is not an important prerequisite for evaluation with parametric tests [25–27]. Therefore, parametric tests are used in the following analysis.

Descriptive data, such as mean values and standard deviations of relevant factors, were calculated. One-way ANOVA was used to evaluate whether the answers of the four groups differed statistically from each other at T0 or T1. This was followed by the calculation of the Tukey post-hoc test to verify which groups exactly differ in their response.

Using the t-test for dependent samples, we assessed whether responses before the demonstration of the materials differed statistically from responses after the demonstration of the materials within one group.

Furthermore, correlations between different items were calculated using Pearson correlation. The Statistical Package for the Social Sciences version 28.0.1 with FixPack 1 for MacOS (SPSS Inc., Chicago, IL, USA) was used for data analysis. The level of significance was set at *p* < 0.05.

## Results

### Sample

One hundred thirteen data sets were collected before the demonstration of the respective material (response rate = 75.3%), and 109 data sets were collected after the demonstration of the material (response rate = 72.7%). Of the surveyed students, 68.4% were female, 31.6% were male and 0% were nonbinary. The arithmetic mean of their age was 25.2 ± 3.3 years.

### Students’ perceived competence levels

**Fig. 2:**
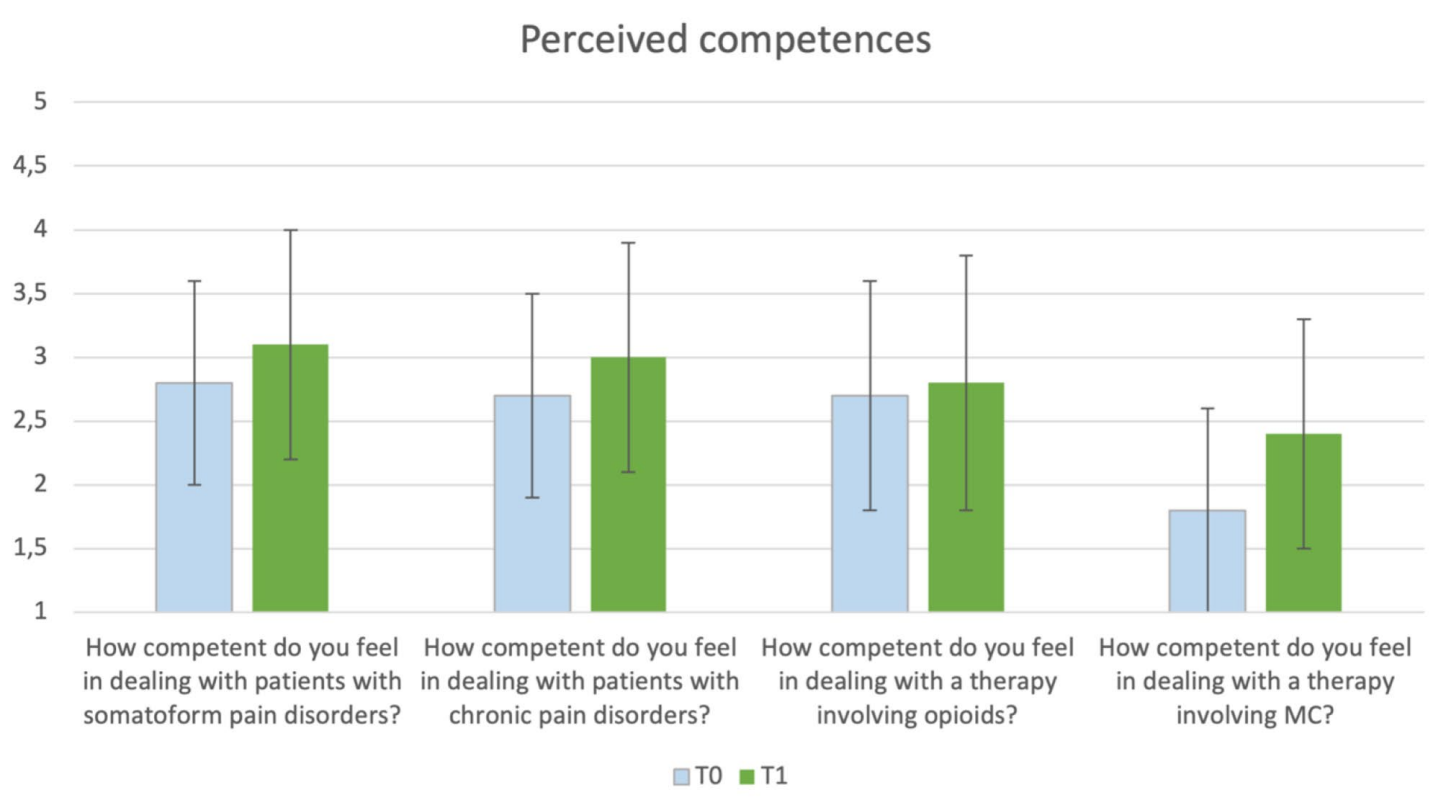
Comparison of the perceived competences at T0 and T1

As shown in Fig. 2, when asked to assess their own competence at T0, the medical students felt only somewhat competent concerning the treatment of patients with chronic pain disorders (M = 2.8 ± 0.8) or somatoform disorders (M = 2.7 ± 0.8). Additionally, they felt significantly more competent regarding therapy with opioids (M = 2.7 ± 0.9) than therapy with MC (M = 1.8 ± 0.8), ${\text{t}}_{0}\left(112\right)=10.5, \text{p}<0.001.$

At T1, the perceived competence increased in all four items. Still, there was a significant difference in the perceived competence regarding therapy with opioids (M = 2.8 ± 1.0) and therapy with MC (M = 2.4 ± 0.9), ${\text{t}}_{1}\left(108\right)=4.4,\text{p}<0.001$.

### Comparison of T0 and T1

#### Differences in perceived competence

**Fig. 3:**
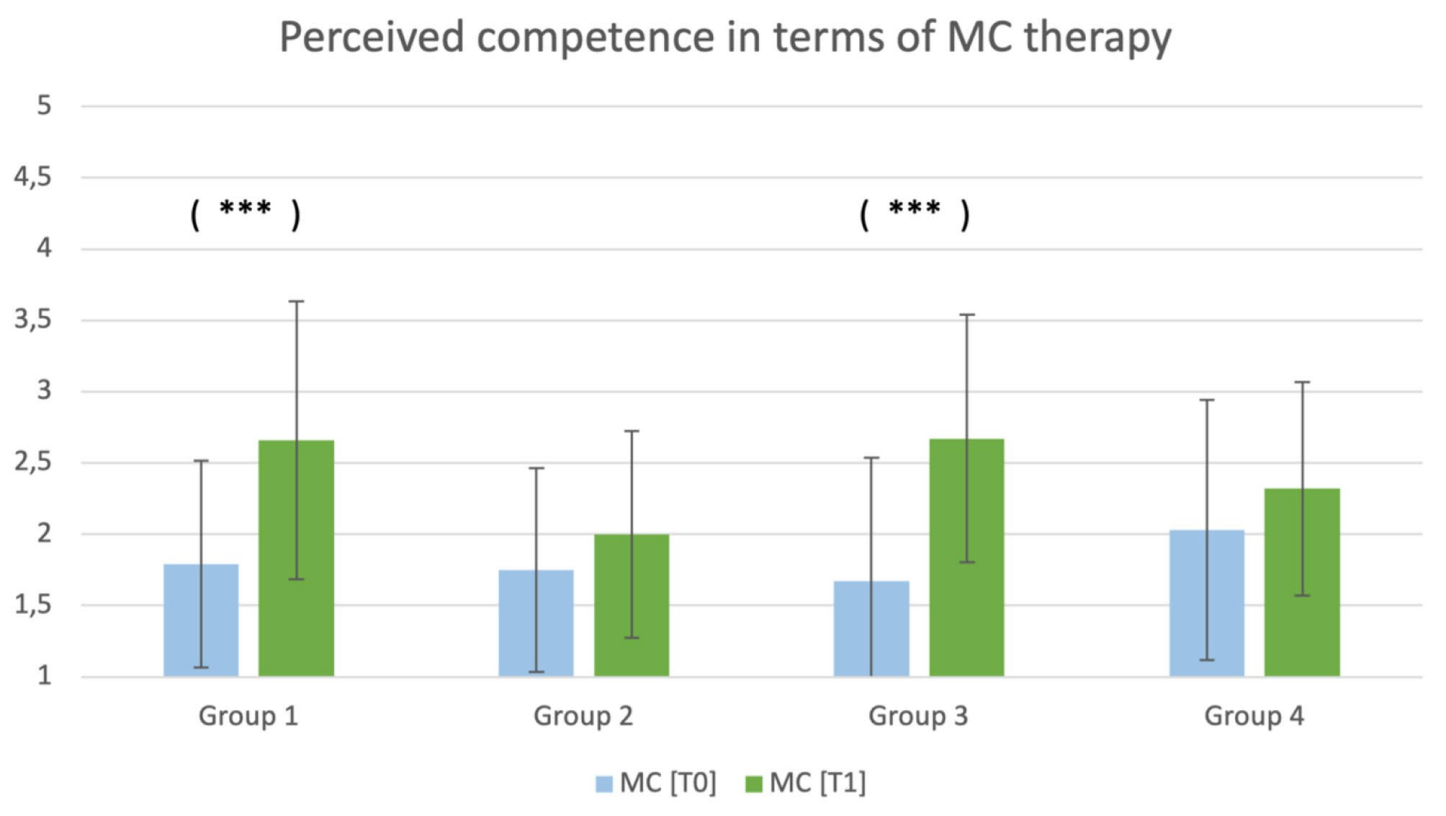
Perceived competence in terms of MC therapy sorted by group at T0 and T1. ***p < 0.001

Greater perceived competence in terms of MC therapy was observed at T1 among the students in group 1 (positive video;$\text{t}\left(28\right)=-3.8,\text{p}<0.001$) and group 3 (positive article; $\text{t}\left(23\right)=-4.2,\text{p}<0.001$), as Fig. 3 shows.

Students in group 3 (positive article) also felt more competent in treating patients with chronic pain disorders at T1 compared to T0 ($\text{t}\left(23\right)=-2.3,\text{p}=0.034$). It was found that students in group 4 (negative article) felt more competent in dealing with patients with chronic pain at T1 ($\text{t}\left(30\right)=-2.0, \text{p}=0.051$).

For the students in group 2 (negative video), there were no significant differences in the perceived competence before and after being shown the negative video. However, they showed greater rejection of the use of opioids at T1 ($\text{t}\left(18\right)=-2.4,\text{p}=0.028$).

### Attitudes and suggestibility– comparison of the four groups

#### At T0

Students in group 1 (positive video) felt more competent than students in group 2 (negative video) concerning the treatment of patients with chronic pain disorders $(0.5;95 \text{\%}-\text{C}\text{I}[0.0;1.1\left]\right)$.

Regarding the statement that cannabis makes a gathering more pleasant, students in group 4 (negative article) were more in favour than students in group 2 (negative video; $-0.9;95 \text{\%}-\text{C}\text{I}\left[-1.6;-0.1\right]$). Students in group 3 (positive article) were more likely to think that teaching about MC should be a part of the medical school curriculum than students in group 1 (positive video; $-0.7;95 \text{\%}-\text{C}\text{I}[-1.2;-0.1])$ and group 2 (negative video; $-0.7;95 \text{\%}-\text{C}\text{I}[-1.2;-0.1])$.

#### At T1

**Fig. 4:**
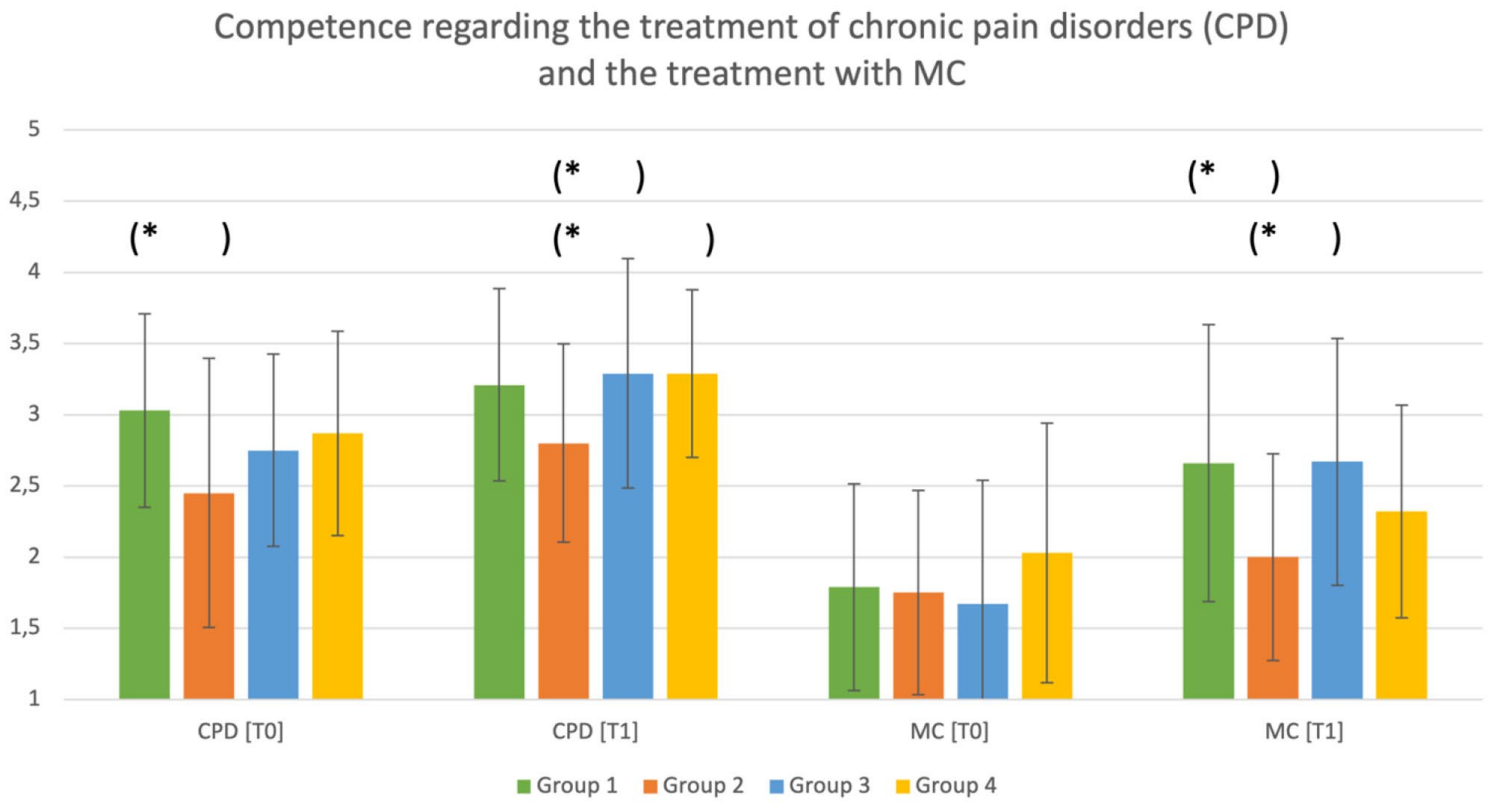
Comparing the perceived competences of the four groups regarding the treatment of chronic pain disorders (CPD) and the treatment with MC at T0 and T1. *p < 0.05

As illustrated in Fig. 4, compared to group 3 (positive article$; -0.7;95 \text{\%}-\text{C}\text{I}[-1.3;-0.1])$ and group 4 (negative article; $-0.7;95 \text{\%}-\text{C}\text{I}\left[-1.4,-0.1\right]$), students in group 2 (negative video) felt significantly less capable of treating patients with chronic pain disorders.

Furthermore, there were significant differences between group 1 (positive video) and 2 (negative video; $0.8;95 \text{\%}-\text{C}\text{I}[0.1;1.4])$ and between group 2 (negative video) and 3 (positive article; $-0.8;95 \text{\%}-\text{C}\text{I}[-1.5;-0.1])$ in relation to handling MC therapy. The students in group 2 (negative video) felt significantly less confident when dealing with MC therapy.

Students in group 2 (negative video) saw fewer advantages for mental health in the consumption of cannabis than the students in group 1 (positive video $;-0.8;95 \text{\%}-\text{C}\text{I}[-1.5;-0.1\left]\right)$ and group 4 (negative video $;-0.7;95 \text{\%}-\text{C}\text{I}[-1.5;0.0\left]\right)$.

In addition, students in group 2 (negative video) favoured the statement that “cannabis can be addictive” less than the other groups. The difference was statistically significant when comparing group 2 (negative video) with group 3 (positive video $;-1.0;95 \text{\%}-\text{C}\text{I}[-2.0;-0.1\left]\right)$.

**Fig. 5:**
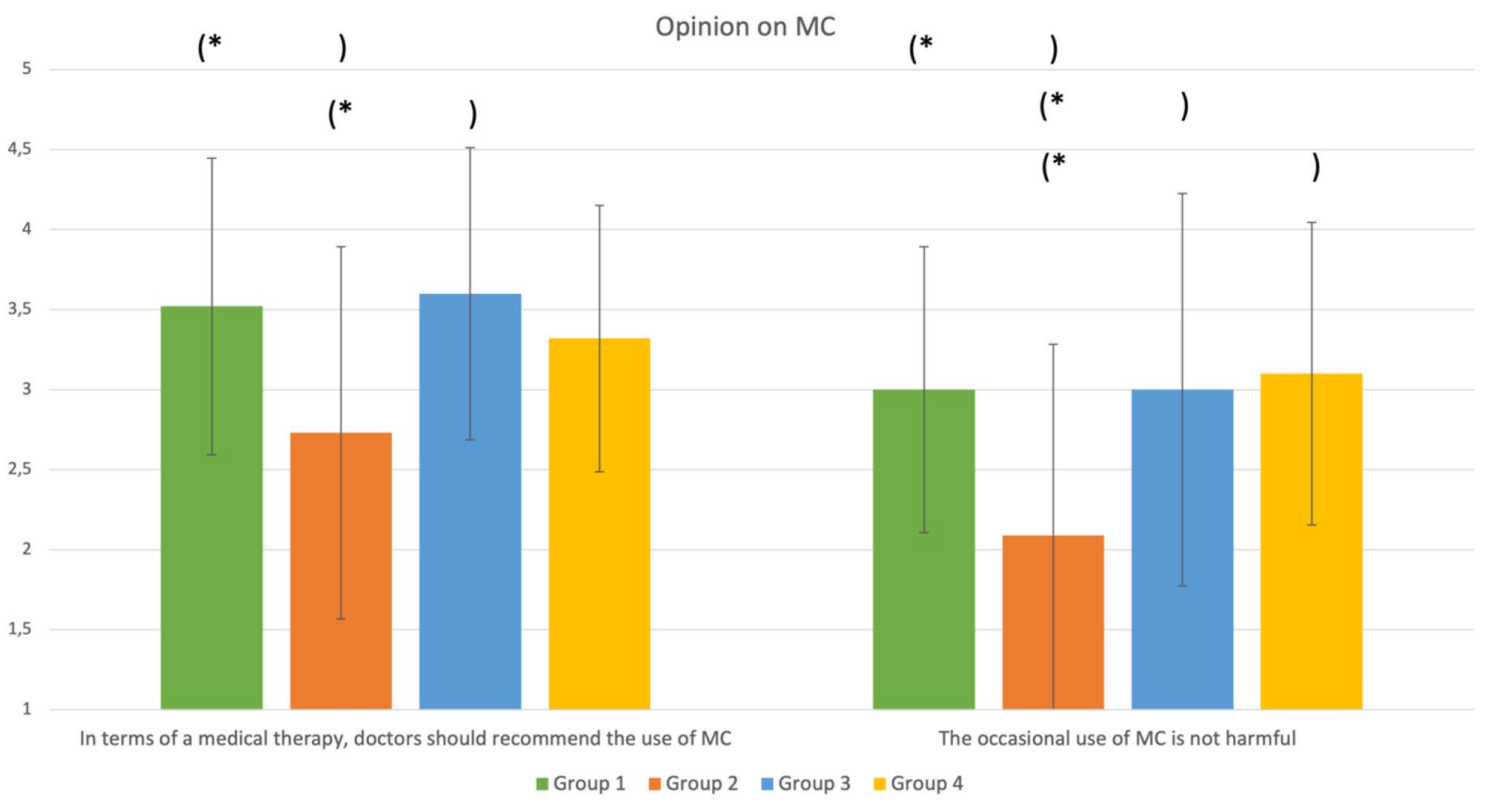
Comparison of the four groups at T1 regarding their opinion on MC. *p < 0.05

As depicted in Fig. 5, students in group 2 (negative video) were more likely to disagree that doctors should recommend the use of MC compared to group 1 (positive video; $\text{0,789};95 \text{\%}-\text{C}\text{I}[\text{0,10};\text{1,48}]$) and group 3 (positive article; $-\text{0,873};95 \text{\%}-\text{C}\text{I}[-\text{1,60};-\text{0,15}]$. Furthermore, students in group 2 (negative video) estimated the occasional use of Cannabis to be more harmful than the students in the other groups: group 1 (positive video; $0.909;95 \text{\%}-\text{C}\text{I}[\text{0,14};\text{1,68}]$), group 3 (positive article; $-\text{0,909};95 \text{\%}-\text{C}\text{I}[-\text{1,71};-\text{0,11}]$) and group 4 (negative article; $-\text{1,006};95 \text{\%}-\text{C}\text{I}[-\text{1,77};-\text{0,24}]$).

Students in group 3 (positive article) were more likely than students in group 2 (negative video) to demand training on MC as part of the medical school curriculum $(0.9;95 \text{\%}-\text{C}\text{I}[0.1;1.8]$). This was also the case regarding the demand for training on MC in continuing medical education $(0.8;95 \text{\%}-\text{C}\text{I}[0.0;1.6]$).

**Fig. 6:**
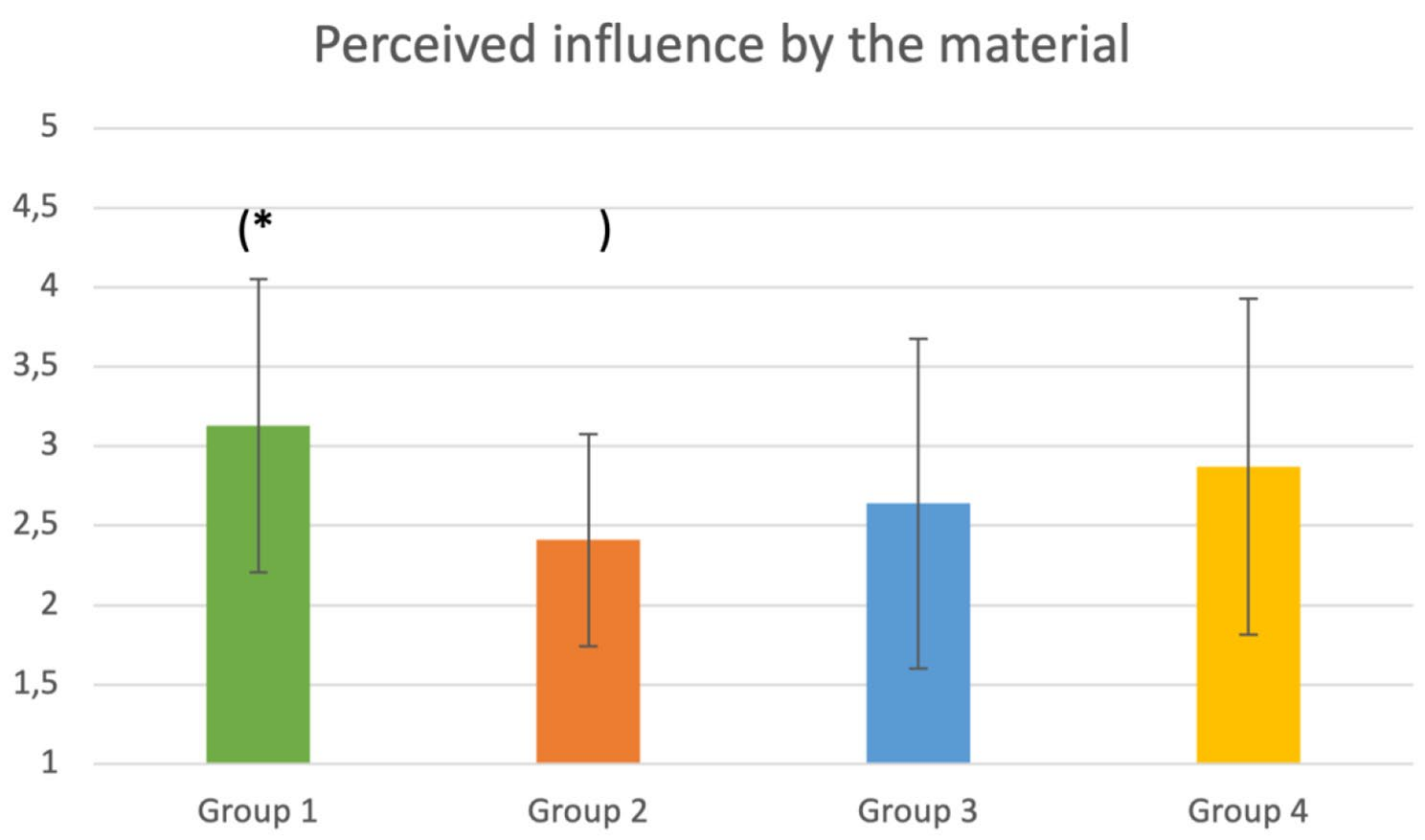
Comparison of the four groups regarding their perceived influence by the material. *p < 0.05

As shown in Fig. 6, students in group 2 (negative video) felt less influenced by the material demonstrated between answering the questionnaires. This observation was significant when compared to group 1 (positive video; $(-0.7;95 \text{\%}-\text{C}\text{I}[-1$0.4;$0.0\left]\right)$.

### Opinions regarding MC and its correlations

At T1, the perceived competence in dealing with MC therapy correlated positively with the perceived competence in educating patients about MC therapy (*r* = 0.62, *p* < 0.001), with the likelihood of this therapy being recommended to a patient (*r* = 0.47, *p* < 0.001), and with the demand that training on MC should be part both in medical school (*r* = 0.34, *p* < 0.001) and continuing medical education (*r* = 0.25, *p* = 0.008).

## Discussion

The aim of this study was to determine the attitudes and perceived competence of medical students towards MC. In addition, the study investigated how students assess their own competence regarding the treatment of patients with chronic pain disorders and how students feel about the use of cannabis and opioids. Furthermore, the study explored how the demonstration of a video or an article (each positive or negative) influences students’ assessment.

### Perceived competence and attitude

The students interrogated felt limited competence regarding the therapeutic use of opioids and MC. Several other studies also came to the conclusion that medical students feel insufficiently prepared to deal with opioids [28, 29] or MC [16, 17, 30, 31]. We showed that the seminar mostly helped improve the students’ perceived competence in dealing with a therapy involving MC, but also in dealing with patients with somatoform or chronic pain disorders. This important finding suggests that there is a strong need for more teaching on MC and opioids in medical schools, and a seminar can improve the students’ perceived competence.

### Need for training

Students clearly demand more training on MC in medical school. This result is underlined by several studies that came to the same finding [16–18, 30, 32]. Furthermore, the demand by medical students for increased training in continuing medical education is significant. A study by Likhitsathian et al. that interrogated medical students in Israel and Thailand came to the same conclusion [30]. Other studies found that medical doctors agree and call for more training through continuing medical education [18, 32–35]. Accordingly, the students’ opinions seem to be transferable to medical professionals. One possible explanation is that until now, teaching about MC has occupied only a small portion of the medical school curriculum. Therefore, students feel insufficiently prepared regarding this subject. This underlines the need for more teaching on MC.

### Influence of the materials presented

About a quarter of the students felt influenced by the material shown during the seminar. A similar finding is described in a study by Kansagara et al. in which doctors were interrogated. It found that 49.4% of the doctors felt influenced by reports about MC via patient reports and 21.6% by the media (e.g. articles) [36].

#### Impact of positive reports about MC on the students’ opinions

Students who were shown materials with positive reports about MC felt more positive towards MC than the other students. This finding is supported by an Israeli study which concluded that people in general can be significantly influenced in their opinions regarding MC through videos [37]. Additionally, after hearing reports from patients who benefitted from using MC, people had a more positive opinion regarding MC [38]. This implies that a sensitive approach should be used when teaching about MC, and one-sided reports about/from patients should be avoided to ensure a differentiated view of the students’ opinions on MC.

#### Impact of a negative patient report on the students’ opinions

The students in group 2 were shown a video sample in which an actor portraying a patient reported his negative experience with MC. After the course, these students were more negative in their opinions towards opioids, felt less competent, rated the mental health benefits of cannabis lower, rated the dependency potential of cannabis higher and, as doctors, would not recommend the use of MC to patients. This confirms a study that concluded that prejudices regarding MC influence the frequency with which nurses and doctors would prescribe MC [39].

In conclusion, we observed that the materials influenced the students in different ways. The greatest effect can be attributed to the negative video report of an actor portraying a patient. The students who were shown this material had a more negative attitude towards MC after the seminar and did not notice a significant increase in their perceived competence.

### Strengths and limitations

As far as we know, this study is the first to explore the influence of different material (videos of an actor portraying a patient or articles) on medical students’ opinions regarding MC. Because attendance was mandatory for the seminar in which the study was conducted, a representative sample of students participated in this research. The large sample size improves the external validity of this study, increasing the likelihood that the results can be generalized to other cohorts / medical faculties. The seminar was conducted according to a standardized procedure, but interposed questions and thus discussions were allowed. The seminar shows one possible approach towards teaching students about MC in pain medicine.

However, this study has some limitations: The articles used as materials for group 3 and 4 are different types of articles (scientific article vs. newspaper article). Hence, their impact on the students’ opinions could vary. However, both articles have reliable sources. The data collected relied on self-reported measures for certain variables, which may be subject to response bias and social desirability effects. The questionnaires at T0 and T1 were not assigned to an individual student. This means that no one-to-one correlation of the questionnaires is possible. Therefore, no intra-individual differences could be calculated. Nonetheless, since we considered an anonymous and general assessment of the students’ perceived competence to be more important, we neglected this point, and the study presents a first impression of how students are influenced by different learning materials regarding MC. Further research should focus on the impact of the legalization of recreational cannabis in Germany. A further study has already been planned to assess medical students’ and physicians’ attitude on this relevant topics by using semi-structured interviews.

### Conclusion

This study investigated the perceived competence of medical students regarding MC, opioids and patients with chronic pain syndrome. Furthermore, the impact of different materials on the students’ opinions was elaborated. The results show that overall perceived knowledge was low but lowest regarding MC. The students who were shown the video of an actor describing his negative experience with MC had a more negative view of MC than the other groups at the end of the course. Simultaneously, students in the other groups demanded more training on MC both in medical school and through continuing medical training. Accordingly, future research should focus on how to adequately educate students and doctors about therapy with MC to make them feel more capable of dealing with it. In addition, there is a need to investigate the extent to which other factors influence the opinions of students and doctors regarding MC. Here, (own) recreational use, age, gender, previous education and other factors could play a role.

## Data Availability

The datasets used and/or analysed during this study are available from the corresponding author on reasonable request.

## Abbreviations

CPD: Chronic pain disorders
DAS: Drug Attitude Scale
MC: Medical cannabis
